# Adjusting line quantum sensing to improve leaf area index measurements and estimations in forests

**DOI:** 10.1016/j.mex.2022.101805

**Published:** 2022-07-30

**Authors:** Guyen Battuvshin, Lucas Menzel

**Affiliations:** Hydrology and Climatology, Institute of Geography, Heidelberg University, Germany

**Keywords:** Beer-Lambert law, Canopy transmittance, Hemispherical photograph

## Abstract

Rapid and reliable estimation of leaf area index (LAI), a crucial parameter in process-based models of vegetation cover response, is important in ecological studies. The Beer-Lambert law is widely used to calculate forest LAI, but data collection methods are time-consuming and calculations are often inaccurate. Our objective was to improve the accuracy of Beer-Lambert law–based LAI estimation by employing indirect data collection and location-specific light extinction coefficients (*K*). Canopy transmittance and LAI of two 100 m^2^ temperate forest stands in southwestern Germany, one managed and one protected, was estimated using line quantum sensing (LQS) at 45,000 points per stand. The Beer-Lambert law was then inverted to estimate LAI using the measured transmittance with a *K* of 0.53–0.54. Hemispherical reference photographs were used as independent validation data to determine ideal *K* values. Experimental data demonstrated that LAI values estimated using LQS with adjusted *K* values were more accurate than those calculated using the basic application of the Beer-Lambert law. LQS results correlated with those determined using hemispherical photography for both the managed (R² = 0.80) and protected (R² = 0.81) stands. Overall, these findings show that adjusting *K* values for individual forest systems improves the accuracy of LAI estimation.

• The modified method is more accurate than that using fixed *K* ranges.
• The modified method accounts for individual ecosystems, with different *K* values for different environments.
• The method can accurately reflect the dynamic changes of forest canopy structure, allowing integration of additional environmental measurements.

## Graphical abstract

**Figure:**
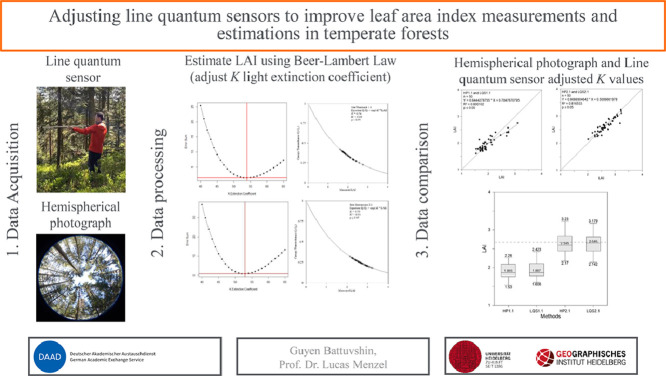

## Specifications table

**Table:** 

Subject Area:	Environmental Science
More specific subject area:	Forestry
Method name:	Leaf area index estimation via line quantum sensing
Name and reference of original method:	Monsi, M. & Saeki, T. On the factor light in plant communities and its importance for matter production. Ann. Bot. 95, 549–567 (2004).
Resource availability:	*not applicable*

## Method background

Leaf area index (LAI) describes the projected leaf area per unit ground area, and is the primary parameter for assessing the structural properties of forests [1]. It is generally accepted that leaves represent the largest portion of the canopy surface, serving as the main site of energy and mass exchange, canopy interception, transpiration, net photosynthesis, and other biological functions. Accurate LAI estimates can provide insight into the scale of these processes [2]. Previous studies have identified LAI as an important variable when characterizing vegetation energy and mass exchange at the macroscopic scale [3]. Furthermore, LAI has been suggested as an important indicator for ecological and environmental pressures on forest ecosystems [4].

In general, LAI can be estimated through direct methods, such as destructive sampling or leaf litter collection [5], or through indirect methods, such as inclined point quadrat sampling [6] or allometric techniques [7]. Direct estimation methods for forest LAI are considered precise and reliable, generally serving as a standard for validating indirect methods [8]. However, they are often time-consuming, labor-intensive, and limited by operational constraints, making direct LAI determination fairly incompatible with long-term monitoring of spatiotemporal leaf area dynamics [9]. Indirect methods, by which leaf area is inferred based on the observations of another variable, are generally faster than direct methods and can be automated, allowing for the acquisition of many samples. In particular, indirect non-contact LAI methods based on measurements of light transmission differences between vegetation and other natural coverage types offer great potential for large-scale spatiotemporal sampling [9].

**Fig. 1:**
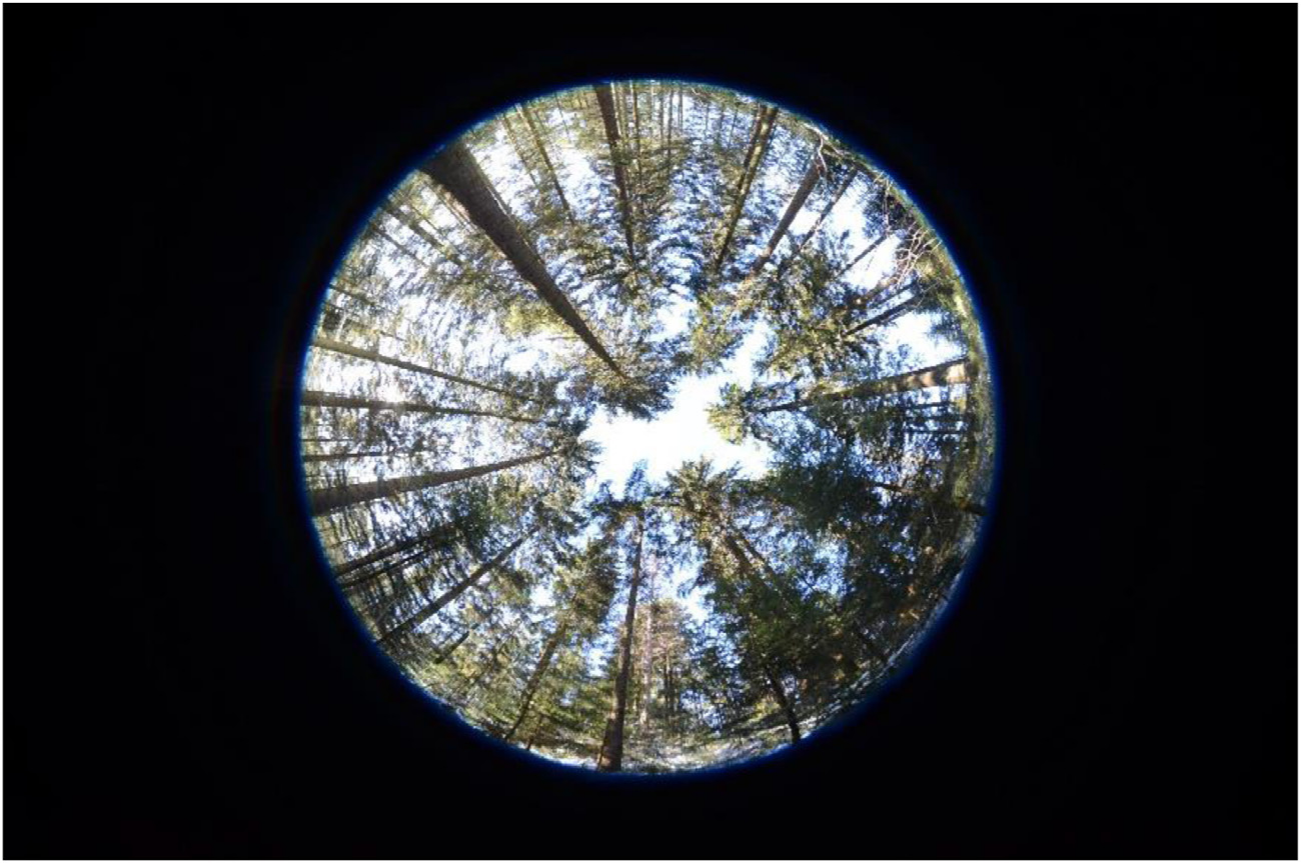
Hemispherical photograph at the Hundseck site, Black Forest, Germany.

Indirect non-contact measurement methods provide real-time LAI information based on the concepts of gap fraction and gap size distribution. Gap fraction analysis compares the differential light measurements above and below the canopy, whereas gap size distribution analysis calculates the percentages of open and canopy-covered areas. Methods such as hemispherical photography (HP), a field-based remote sensing approach used to characterize biophysical canopy structure via light attenuation and contrast measurements, use image analysis to determine gap size distribution and develop permanent records of forest cover (Fig. 1). HP is a valuable source of information regarding the position, size, density, and distribution of canopy gaps, and can capture species-, site-, and age-specific characteristics of canopy structure with a 180° viewing angle [6]. HP is considered a powerful indirect method for measuring various components of canopy structure, but cannot be performed under direct sunlight, which causes distortion of images (i.e., over-exposure in a certain image region). Therefore, it may lead to underestimates of LAI and cannot be used at all times of day. Methods based on gap fraction analysis, such as a line quantum sensing (LQS), involve ground-based measurements of the total, direct, and diffuse radiation transmittance to the forest floor. Light interception can be measured above the canopy or, in the case of tall stands, in a nearby open area [10]. Such methods consider that the total amount of radiation intercepted by a canopy layer depends on incident irradiance, canopy structure, and optical properties [11], and rely on the Beer-Lambert law to estimate LAI. This law describes the classic relationship between LAI and gap probability [12,13], and is expressed as:

(1) $$I=I_{0}*\;e^{-K\left(LAI\right)}$$

Eq. (1) was transformed to:

(2) $$LAI=\frac{-ln\left(\frac{I}{I_{0}}\right)}{K},$$

where LAI is the projected leaf area index, I is average below-canopy photosynthetically active radiation (PAR), $I_{0}$ is the average total incoming PAR, and *K* is the light extinction coefficient [11]. This equation assumes that the LAI normal to a radiation beam is independent of the angle at which the radiation is incident on the canopy, that leaf inclination angles are spherically and randomly distributed, and that plant leaves are distributed randomly in space [14].

Recently, LAI estimation methods using the Beer-Lambert extinction law have been utilized by large-scale forest monitoring agencies, and are favored for industrial and research applications because they provide a reliable description of average canopy conditions [11,15]. However, these methods require the selection of a *K* value, which is challenging because of the potential range in solar radiation caused by cloud cover. A proper *K* must be site- and species-specific, and should account for leaf angle, form, and clumping [16]. Pierce and Running [17] proposed the use of a constant *K* value of 0.52 for coniferous species, based on measurements reported by Jarvis and Leverenz [14]. However, the *K* inherently depends on stand structure and canopy architecture, meaning that a specific *K* should be selected for any given stand [18].

Despite clear advantages of methods such as LQS, including speed and reproducibility, *K*-based estimates are limited by variability, and studies have not effectively verified their accuracy compared to that of more established methods. For example, investigations requiring fine details of canopy structure (i.e., angular distributions of foliage) or light penetration (i.e., bidirectional gap fraction), HP is advantageous owing to its spatial discrimination [7]. In other studies, however, LQS has been found to estimate the LAI of crops and forests more accurately than other methods [19]. LAI estimates made by HP and by LQS have not been comprehensively compared in structurally complex environments, such as protected and managed temperate coniferous forest. Furthermore, it is not clear which *K* can be reliably used in these types of forest.

In this study, LQS-based LAI values were calculated for two temperate coniferous forest stands and compared against those obtained using the standard HP method. Additionally, the accuracy of LQS-based LAI values in relation to different *K* was investigated. The primary objectives of this study were (1) to determine if transmittance measurements obtained using LQS can provide accurate estimates of LAI in a temperate coniferous forest, and (2) to analyze how differing stand structures (managed vs protected forests) and *K* affect measurement accuracy. We aimed to improve the accuracy of the LQS technique by correcting raw quantum sensor data collected under heterogeneous light conditions. Our findings show that use of stand-specific *K* can overcome the limitations of LQS-based LAI estimations.

## Method details

### Study area

The study was conducted in the Black Forest of southwestern Germany, a forested low mountain range at an average sea level of 800–1500 m (Map 1). The region is surrounded by the Rhine River valley to the west and the south, and has a length of approximately 160 km and an average width of 50 km. Like other low mountain ranges in Central Europe, the Black Forest is considered an ecologically important yet vulnerable area due to increasing threats of drought [19] and reduced snow cover [20]. The mean temperature is 4–6 °C, and the mean annual precipitation is 1700 to 2000 mm, depending on the altitude [21]. The dense and extensive forests comprise 80% coniferous (spruce, fir, and pine) and 20% deciduous (beech, birch, and oak) tree species [22]. Our study sites were located at Hundseck (site code 1.1) and Herrenwies (site code 2.1) in the northern Black Forest (Table 2).

**Fig. 2:**
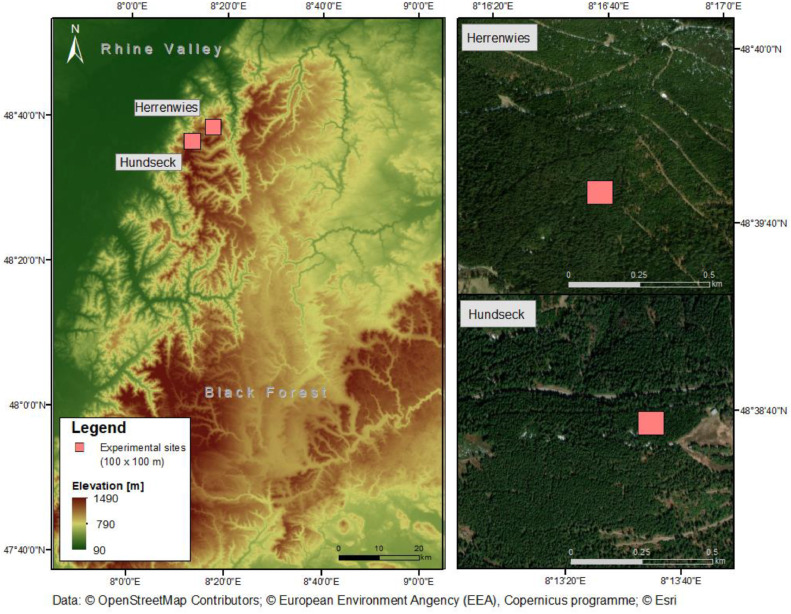
Overview of the two study areas, Hundseck and Herrenwies, in the Black Forest, Germany. Colored rectangles indicate the locations of the experimental plots. Data origin: BKG, 2020 and Openstreetmap (gdz.bkg.bund.de; planet.openstreetmap.org).

Based on our manual measurements, tree density, average tree height, and average tree diameter were approximately 0.8/100 m², 22 m, and 97 cm, respectively. The climatological data of the study areas were obtained from a meteorological station located near the study sites (<1 km) [23]. LAI was measured in sections of forest stands of 100 m² in size, which were chosen as representative stands for the Black Forest based on their typical variety of structures and species compositions (Fig. 2).

### LQS data collection and processing

The canopy transmittance of each stand was sampled using an LI-191R Line Quantum Sensor (LI-COR Biosciences, Lincoln, NE, USA), a handheld device designed to measure light in photosynthetic photon flux density (µmol s^−1^ m^−2^) units. The sensor contained a light-sensitive 1 m quartz rod under a diffuser to direct light to a single filtered silicon photodiode, integrating measurements from an essentially infinite number of points over its surface into a single representative value. The device was attached to a battery-powered digital data logger (model LI-250A/Light Meter, LI-COR), which provided a direct digital readout and displayed instantaneous averages of values taken every 15 s (approximately 60 readings) to instantly calculate PAR. Readings were recorded in 2020 and 2021 (Table 1) on the same day and under the same conditions as those of the HP data collection to minimize any bias when comparing the results.

**Table 1:** Dates of photographs and LQS measurements.

Site name	Longitude	Latitude	Altitude a.s.l (m)	Main tree species	Photographs and LQS measurements			
					2020			2021
Managed forested stands								
Hundseck (1.1)	08°13′35′E	48°38′33′N	929	*Picea abies*	May.13/ Jun.07	Aug.10/ Aug.30	Dec.03	Feb.04
Protected forested stands								
Herrenwies (2.1)	08°16′30′E	48°39′50′N	784	*Picea abies*	May.13/ Jun.07	Aug.10/ Aug.30	Dec.03	Feb.04

Diffusion below the canopy was sampled along a systematically gridded transect with a random starting point. Each transect contained 50 sample stops spaced approximately 14 m apart. At each stop, PAR measurements were obtained by holding the quantum sensor with outstretched arms and turning a full 360°. The measurements were manually recorded and averaged. At each stop, the effective sample area was approximately 6 m². The full transect took less than 1 h to complete and represented 45,000 PAR sampling points per stand. Total incoming PAR was measured at the beginning and end of each transect in a nearby clear-cut or open area. When possible, additional measurements of the total incoming PAR were made in large (>100 m²) canopy openings during sampling.

To find the best-fitting *K* value for each stand, LAI was estimated using Eq. (2) with *K* values ranging from 0.40 to 0.65. Then, these estimated LAI values were compared against HP-based independent validation data using Eq. (3). Analyses were performed in RStudio (RStudio Team, 2020; see “Statistical analysis” below). Finally, assuming that necessary assumptions for the Beer-Lambert law with adjusted *K* were met by LQS measurements, Eq. (2) was transformed into Eq. (4) and transmittance for each stand based on measured LAI with adjusted *K* values.

### HP data collection and processing

Photos were taken using a digital camera (D7000, Nikon, Tokyo, Japan) with a 180° grade fisheye lens (EX-DC, Sigma, Kawasaki, Japan), mounted on a tripod with manual levelers (Dörr Airpod, Germany). All HP images were visually assessed for overexposure and blooming effects to select for optimal representation of the canopy. The selected photos were analyzed using Hemisfer 2.2 (Swiss Federal Institute for Forest, Snow, and Landscape Research) to estimate the LAI and light regime based on published methods [4]. Classification of each pixel as sky (white pixels) or canopy (black pixels) was conducted based on the brightness threshold values of the red, green, and blue color channels present in the photographs. The color frequencies were calculated and graphically assessed to automatically set the appropriate threshold according to the algorithm described by Schleppi et al. [4]. The algorithm was then used to transform the photos into binary black and white images. The images were searched for borders, which were identified as areas having the steepest color gradients between neighboring pixels for any color combination [4]. Sky was defined as white pixels, and canopy by black. LAI was then estimated using a published method [24].

### Statistical analysis

Based on the LAI estimated by LQS, with *K* values in the range of 0.40–0.65, a *K* value for each managed and protected forest stand was calculated using the following equation:

(3) $$\text{Sum}\;\text{of}\;\text{squares}=\sum_{i=1}^{n}{\left(X_{LQS}-\;{\bar{X}}_{HP,\;i}\right)}^{2},$$

where $X_{LQS}$ is the LQS-based LAI, ${\bar{X}}_{HP,\;i}$ is the HP-based LAI, and $X_{LQS}-\;{\bar{X}}_{HP,\;i}$ is the difference between the LQS and HP values. Therefore, Eq. (3) yields the summed difference between HP and LQS-based LAI calculated with given *K* value. The sum of squares statistic was determined as described by Allen [25]. The range of variation was described as a percentage of the LAI such that, at given sample point, one could determine the error contributed by the sampling technique. Given the validity of the Beer-Lambert Law assumptions, we transposed Eq. (2) to predict the transmittance for each stand based on the measured LAI and adjusted *K*:

(4) $$\frac{I}{I_{0}}=\exp\left(-K*LAI\;\right)$$

where $I/I_{0}$ is canopy transmittance, K is the light extinction coefficient, and LAI is the estimated LAI via the LQS. Measured canopy transmittance was plotted against measured LAI and adjusted *K* for each stand to observe statistical correlations (Figs. 8 and 9). LAI values obtained using HP and LQS methods were compared using three statistical tests, including the Kolmogorov-Smirnov test [26], the Wilcoxon test [27], and linear regression [28].

## Method validation

**Fig. 3:**
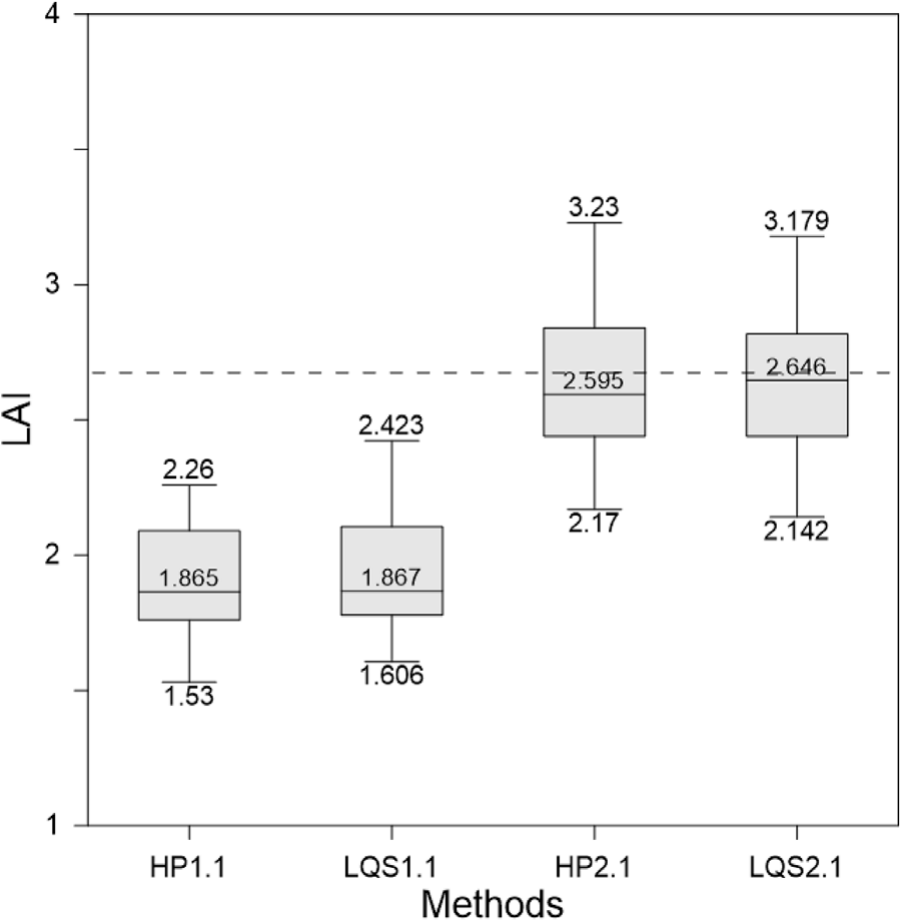
Comparison of the leaf area index (LAI) values obtained from hemispherical photography (HP) and line quantum sensing (LQS) with the average global LAI for temperate forests (dashed line; from [29]). The number following each abbreviation represents the site code (x-axis, sites at Hundseck, 1.1, *n* = 50; Herrenwies, 2.1, *n* = 50).

**Fig. 4:**
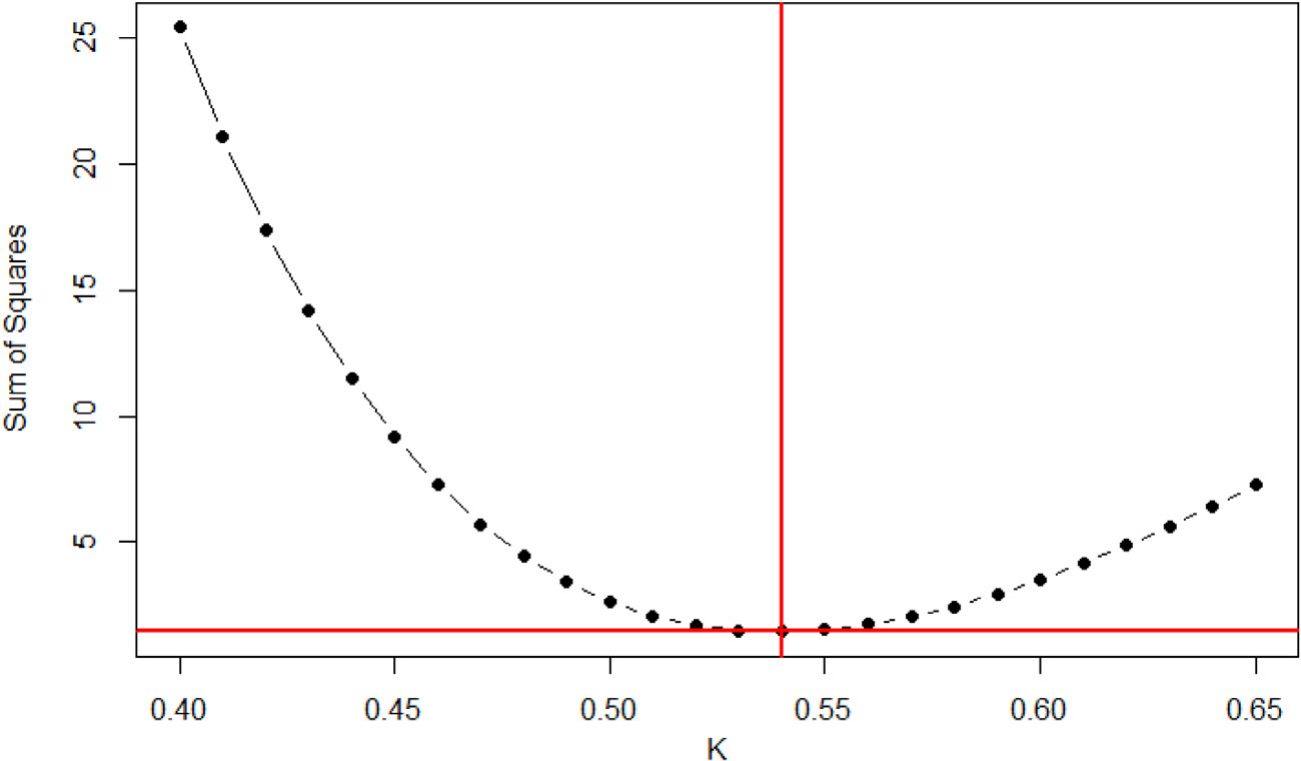
Comparison of *K* (0.40–0.65) used to process line quantum sensor readings at Hundseck. Based on the test result (*K* = 0.54), the leaf area index was estimated and compared to hemispherical photograph values, Fig. 6.

**Fig. 5:**
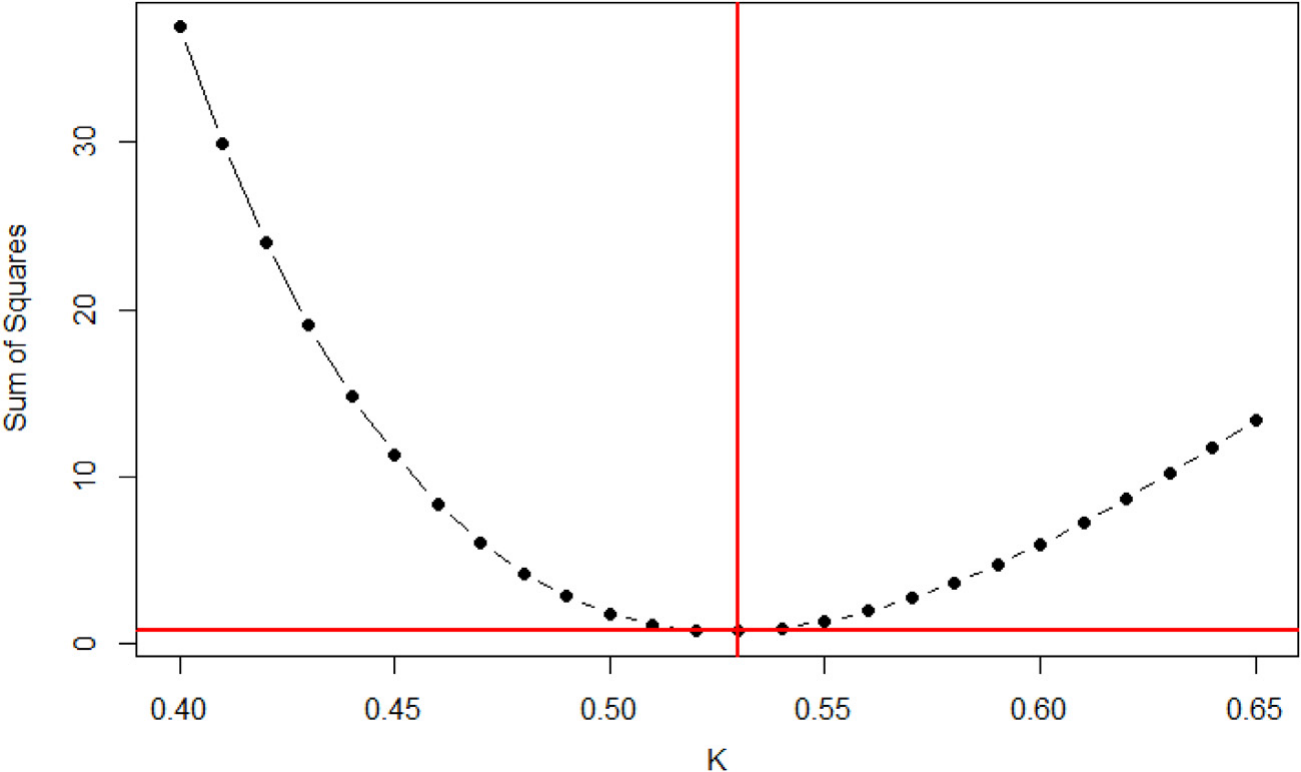
Comparison of *K* (0.40–0.65) used to process line quantum sensor readings at Herrenwies. The leaf area index was estimated based on test results (*K* = 0.53) and compared to hemispherical photograph values, Figs. 8 and 9.

A comparison of the HP and LQS LAI values is presented in Fig. 3. The LQS values were calculated through Eq. (2) using our adjusted *K* for managed and protected temperate coniferous forests, respectively. Figs. 4 and 5 show the LAI value derived from the LQS readings tested through the *K* ranges (0.40–0.65) using Eq. (3) for Hundseck. The *K* value demonstrated a convex curve along with the differences between HP and LQS-based LAI, first decreasing and then increasing. Based on our findings for both managed and protected forests, *K* values from 0.40 to 0.52 indicated an overestimated LAI value, while those in the 0.55 to 0.65 range indicated an underestimated LAI value. In addition, it was also found that change of *K* value in the same variety was different than that between varieties of managed and protected forest management types. The probable explanation was that along with the management of the forest, the number of young trees lower compared to protected forest. The resulting *K* = 0.54, was considered appropriate value for Hundseck, and *K* = 0.53 for Herrenwies.

**Fig. 6:**
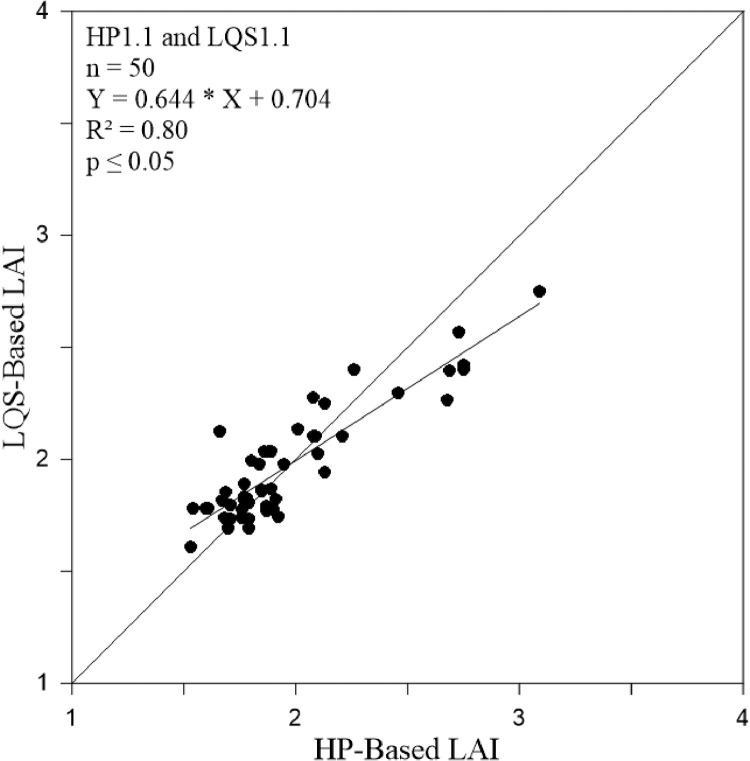
Linear regression of leaf area index (LAI) values (site code 1.1) from the hemispherical photography (HP) and line quantum sensing (LQS) readings calculated with a *K* of 0.54.

**Fig. 7:**
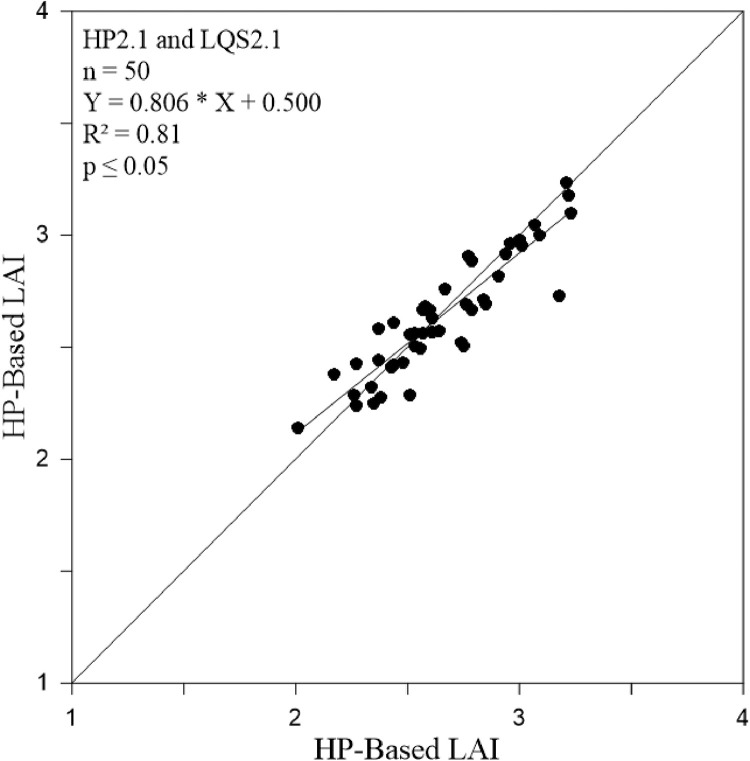
Linear regression of the leaf area index (LAI) values (site code 2.1) from the hemispherical photography (HP) and line quantum sensing (LQS) readings calculated with a *K* of 0.54.

**Fig. 8:**
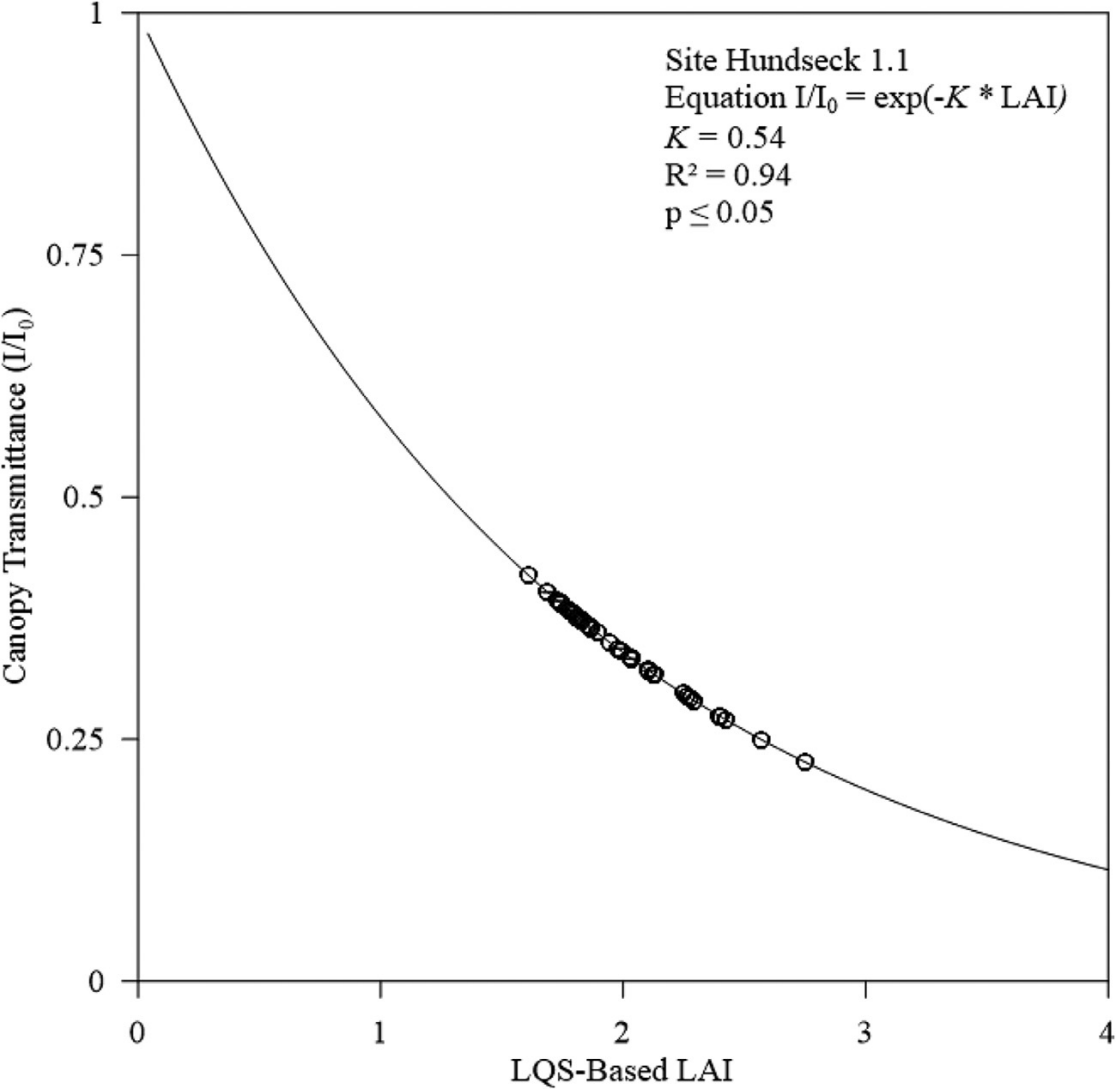
Relationship between stand projected leaf area index (LAI) and canopy transmittance $\left(I/I_{0}\right)$ measured at Hundseck (1.1). The Beer-Lambert law defines the curve passing along the data points; *K* *=* 0.54.

**Fig. 9:**
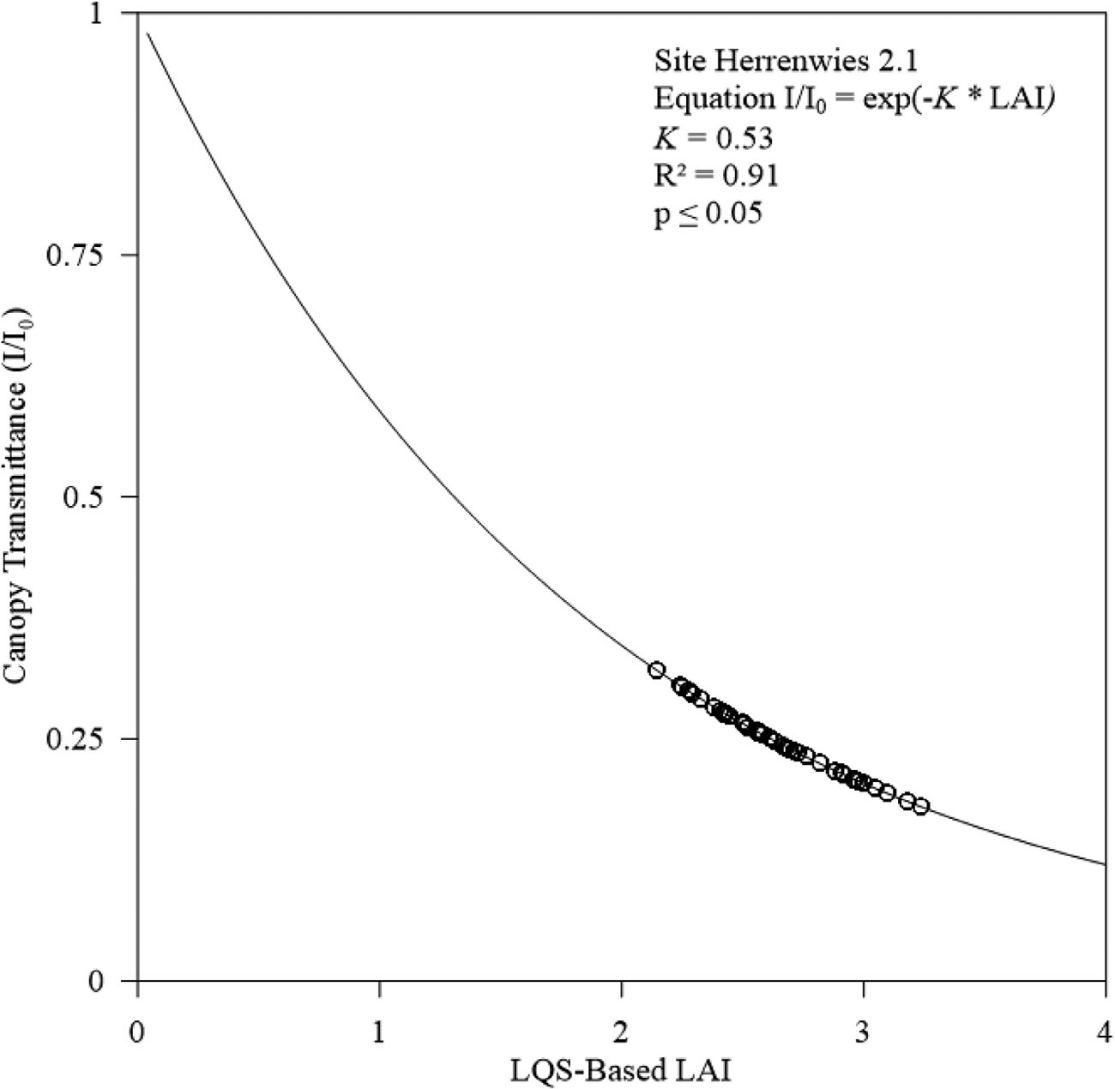
Relationship between stand projected leaf area index (LAI) and canopy transmittance $\left(I/I_{0}\right)$ measured at Herrenwies (2.1). The Beer-Lambert law defines the curve passing along the data points; *K* = 0.53.

To further support our adjusted *K* values for temperate coniferous forest, we statistically tested and compared HP and LQS-based LAI values calculated using adjusted *K* (Figs. 6 and 7) and R² values at Hundseck and Herrenwies (0.80 and 0.81, respectively). The result indicated a strong correlation. Furthermore, the statistical test values showed strong relationships (Table 2). As shown in Figs. 8 and 9, we transposed Eqs. (2)–(4) to predict the transmittance for each stand based on measured LAI and adjusted *K* values, then plotted a curve through the data points. Transmittance values were not significantly different between stands, with R² values of 0.80 (*p* ≤ 0.05) at Hundseck and 0.81 at Herrenwies (*p* ≤ 0.05).

**Table 2:** Leaf Area Index (LAI) values obtained through two indirect survey methods, statistical methods: (KS) Kolmogorov-Smirnov test, (W) Wilcoxon test, and (Lm) Linear models.

Site code	*n*	LAI						Statistical methods		
		Hemispherical photograph			LQS			KS	W	Lm
		Min	Max	Median	Min	Max	Median	p - value		
Managed forested area										
Hundseck (1.1)	50	1.53	2.26	1.865	1.606	2.423	1.867	0.864	0.86	≤0.05
Protected forested area										
Herrenwies (2.1)	50	2.17	3.23	2.595	2.17	3.179	2.646	0.54	0.43	≤0.05

## Conclusions

We used LQS to measured canopy structure parameters in managed and protected temperate coniferous forests. We then applied a modified Beer-Lambert law calculation to select site-specific *K* values, improving the accuracy of LAI estimation. One of the main uncertainties was the estimation of *K* in individual ecosystems because of values range from 0.40 to 0.65. Because *K* values can significantly influence LAI estimates obtained using LQS, values should be adjusted for different forests to avoid over- or underestimation. Few studies have assessed LAI by adjusting *K* values for differently managed forests. Our results suggest that forest management has a certain influence on LAI and *K* values. In this study, the *K* value of the protected forest (0.53) tended to be lower than that in the managed forest (0.54). Furthermore, referencing to true ground data, such as HP measurements, is important for selecting the most accurate value. This study demonstrates the use of adjusted *K* for LQS-based LAI measurements in temperate coniferous forest stands, which can be applied to various ecological and environmental monitoring projects.

This method exhibited high performance compared with that of HP-based analysis, although some limitations were noted, many of which have been discussed by Jonckheere et al. [7] and Tan et al. [15]. In particular, the present study focused only on transmittance above and below the canopy, without exploring the vertical distribution of structural parameters along various layers of the forest canopy. Moreover, transmittance information from LQS is obtained in the direction of the vertical canopy of the forest.

We found that LQS should be conducted under clear weather conditions to prevent water droplets from interfering with the sensors. To ensure accurate, representative LQS readings, the instrument should be rotated 360° at a constant speed, and should be held with an outstretched arm at chest level to avoid interference from the shadow of the body, which could be misidentified as vegetation cover and lead to false LAI estimation. Averaging the readings from a 360° rotation may improve the accuracy of LQS-based LAI estimation. LQS-based LAI estimation is more cost-effective and less time-consuming than manual or automatic observation methods. Combined with site-specific *K* values, it offers potential for establishing intensive monitoring networks, particularly in areas with complex environmental conditions.

By adjusting *K* values to increase the accuracy of LAI estimation via LQS, we introduced an innovative and accurate technique for assessing structural information between the horizontal forest canopy space and the ground. Furthermore, our methods allowed for specific monitoring of differently managed forests based on LAI with adjusted *K* values, improving estimation accuracy compared to the original method with fixed *K* values. Our findings address current limitations in data collection and forest monitoring, showing a distinctive improvement to estimation accuracy by adjusting *K* values for specific sites (0.40–0.65). These insights will facilitate the development of novel forest protection and management methods, supporting ecosystem monitoring overall.

## Declaration of Competing Interests

The authors declare that they have no known competing financial interests or personal relationships that could have influence the work reported in this paper.
